# Characterizing Dynamic Interactions between Ultradian Glucocorticoid Rhythmicity and Acute Stress Using the Phase Response Curve

**DOI:** 10.1371/journal.pone.0030978

**Published:** 2012-02-21

**Authors:** James Rankin, Jamie J. Walker, Richard Windle, Stafford L. Lightman, John R. Terry

**Affiliations:** 1 Bristol Centre for Applied Nonlinear Mathematics, University of Bristol, Bristol, United Kingdom; 2 INRIA Sophia-Antipolis, Sophia Antipolis, France; 3 Henry Wellcome Laboratories for Integrative Neuroscience & Endocrinology, University of Bristol, Bristol, United Kingdom; 4 School of Nursing, Midwifery and Physiotherapy, University of Nottingham, Nottingham, United Kingdom; 5 Department of Automatic Control and Systems Engineering, University of Sheffield, Sheffield, United Kingdom; 6 Sheffield Institute for Translational Neuroscience, University of Sheffield, Sheffield, United Kingdom; University of Texas Health Science Center at San Antonio, United States of America

## Abstract

The hypothalamic-pituitary-adrenal (HPA) axis is a dynamic oscillatory hormone signalling system that regulates the pulsatile secretion of glucocorticoids from the adrenal glands. In addition to regulation of basal levels of glucocorticoids, the HPA axis provides a rapid hormonal response to stress that is vitally important for homeostasis. Recently it has become clear that glucocorticoid pulses encode an important biological signal that regulates receptor signalling both in the central nervous system and in peripheral tissues. It is therefore important to understand how stressful stimuli disrupt the pulsatile dynamics of this system. Using a computational model that incorporates the crucial feed-forward and feedback components of the axis, we provide novel insight into experimental observations that the size of the stress-induced hormonal response is critically dependent on the timing of the stress. Further, we employ the theory of Phase Response Curves to show that an acute stressor acts as a phase-resetting mechanism for the ultradian rhythm of glucocorticoid secretion. Using our model, we demonstrate that the magnitude of an acute stress is a critical factor in determining whether the system resets via a Type 1 or Type 0 mechanism. By fitting our model to our *in vivo* stress-response data, we show that the glucocorticoid response to an acute noise stress in rats is governed by a Type 0 phase-resetting curve. Our results provide additional evidence for the concept of a deterministic sub-hypothalamic oscillator regulating the ultradian glucocorticoid rhythm, which constitutes a highly responsive peripheral hormone system that interacts dynamically with hypothalamic inputs to regulate the overall hormonal response to stress.

## Introduction

**Figure 1:**
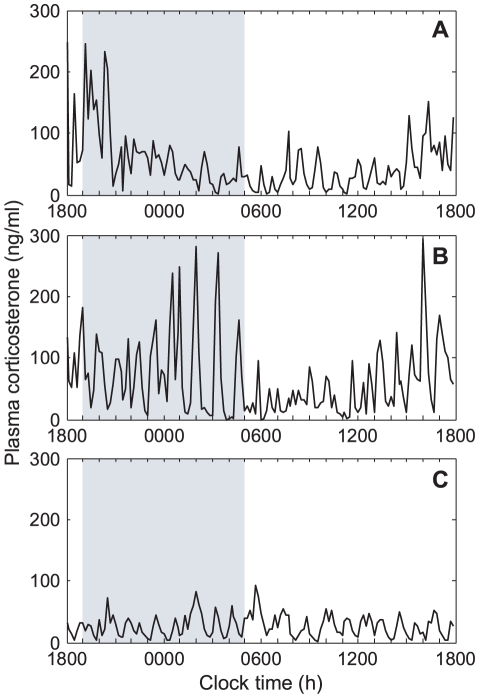
Ultradian glucocorticoid oscillations in physiological and pathophysiological states. (A–B) In basal (unstressed) conditions, diurnal variation in hormone levels is not smooth but is reflected by a circadian modulation of ultradian pulse amplitude. Data shown is from female Sprague-Dawley rats (A) and female Lewis rats (B). (C) In male Piebald-Viral-Glaxo (PVG) rats with chronic inflammatory stress, pulse amplitude during the circadian nadir is comparable to pulse amplitude during the circadian peak. Shaded region indicates the dark phase.

The hypothalamic-pituitary-adrenal (HPA) axis regulates levels of circulating glucocorticoid hormones (CORT–cortisol in humans, corticosterone in rodents), which in turn mediate a wide range of physiological processes, including metabolic, immunological and cognitive function [1]. The activity of the HPA axis follows a distinctive circadian pattern of activity with low glucocorticoid levels during the resting period, which increase to a peak around the time of wakening. Underlying this circadian rhythm, however, is a highly dynamic ultradian rhythm (near hourly oscillations) of glucocorticoid release (Figure 1). It is now clear that glucocorticoid pulsatility is important for dynamic transcriptional regulation of target genes in both the liver and the brain [2], [3], and these pulses therefore form the basis for an extremely rapid and sensitive hormone signalling system [4]. Whilst the pulsatile secretion of glucocorticoids has traditionally been assumed to result from the activity of neural pacemakers within the hypothalamus [5], [6], more recent theoretical findings suggest that the ultradian rhythm may in fact be regulated by pituitary-adrenal interactions, independent of pulsatile hypothalamic activity [7], [8].

**Figure 2:**
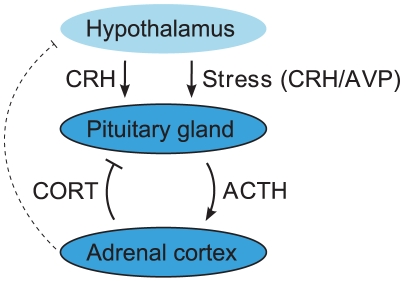
Schematic of the HPA axis. Under basal conditions, the pituitary gland releases readily-available ACTH in response to CRH secreted from the hypothalamus. In response to ACTH, the adrenal cortex synthesizes and secretes CORT, which feeds back primarily at the pituitary gland, but also at higher centres in the brain (depicted by the dashed line), to inhibit ACTH secretion. This dynamic balance between the positive feed-forward action of CRH and the negative feedback action of CORT at the level of the pituitary gland has been suggested theoretically to regulate the oscillatory activity of the pituitary-adrenal system. In addition to basal regulation, acute stressors result in additional hypothalamic secretion (CRH and AVP) which act to further stimulate the pituitary-adrenal network. The interplay between basal oscillatory activity and acute stress perturbations is the focus of this study.

In addition to regulating basal glucocorticoid pulsatility, the HPA axis is an important component of the mammalian response to stress [9]. Cognitive stressors, as well as more physical stressors like inflammation or hypotension, activate neurons in the paraventricular nucleus (PVN) of the hypothalamus to release corticotrophin-releasing hormone (CRH) and arginine vasopressin (AVP) into the portal circulation, which in turn stimulate adrenocorticotropin (ACTH) release from corticotroph cells in the anterior pituitary. ACTH is then transported via the circulation to the adrenal cortex where it activates glucocorticoid synthesis and release, which subsequently feeds back on the corticotroph cells to inhibit further ACTH release (Figure 2). Consequently, a stress–and its associated release of CRH/AVP–can be considered as a perturbation to endogenous system activity. Given that many biological systems are regulated by both positive and negative feed-forward and feedback loops, which in turn permits dynamic oscillatory signalling, this makes the HPA axis an interesting model system to explore how exogenous system perturbations interact with endogenous oscillatory activity.

In previous experimental studies where the HPA response to an acute stress in freely-behaving rats was investigated, the timing of the stress relative to the phase of the underlying ultradian rhythm was shown to be crucial in determining the magnitude of the corticosterone response [10]–[12]. In particular, when the stress coincided with the rising (secretory) phase of the ultradian rhythm, corticosterone concentrations rose markedly. In comparison, a stress coincident with the falling phase resulted in a less marked hormonal response. Given the importance of glucocorticoid pulsatility, in this study we perform a more quantitative characterisation of the interaction between acute stress and the endogenous glucocorticoid ultradian rhythm.

## Materials and Methods

### Subjects

The data here have appeared in three previous studies [10]–[12], where details of experimental methods were described in detail. We therefore provide only a brief summary of the relevant experimental procedures. In each of these earlier studies, experiments were carried out using a different strain of rat: female Sprague-Dawley (SD) [10], female Lewis [11], and male Piebald-Viral-Glaxo (PVG) [12]. All animals were maintained under standard animal housing conditions (14 h light, 10 h dark illumination cycle with lights on at 0500 h).

### Surgery

In all three studies [10]–[12], the right jugular vein of anaesthetized animals was exposed, and a cannula was inserted into the vessel. The free end of the cannula was exteriorized through a scalp incision and then tunnelled through a protective spring that was anchored to the parietal bones. Animals were then individually housed and the springs were attached to freely-rotating mechanical swivels, which provides the animals with maximum freedom of movement. Animals were given a recovery period of 5 days after surgery.

### 24 h corticosterone profiles

Cannulae were connected to an automated blood sampling (ABS) system and samples were collected at 10 min intervals for 24 h beginning at 1800 h. In [12], the male PVG rats were pretreated (13 days) with an intradermal injection (0.1 ml) of a suspension of ground, heat killed *Mycobacterium butyricum* in paraffin oil (10 mg/ml) into the base of the tail to induce arthritis.

### Corticosterone responses to noise stress

Blood samples were collected at 10 min intervals beginning at 0600 h. After a period of basal activity (120–140 min), a white noise generator was activated, and the rats were exposed to 114 decibels (12,000–60,000 Hz) for 10 min. Sampling continued for 120–240 min after the stress.

### Hormone measurement

Total plasma corticosterone concentrations were measured directly in plasma by radioimmunoassay (RIA) as previously described [10]–[12].

### Numerical model and simulations

To investigate the dynamical behaviour of this system theoretically, we used a systems-level model of the HPA axis which we introduced in [7] and is based in part on the work of Gupta *et al.* [13]. This model was developed *independently* of the experimental data within this study and is based solely on the known feed-forward and feedback interactions between that anterior pituitary and adrenal glands. The model (in dimensionless form) is a system of delay differential equations (DDEs):
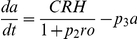


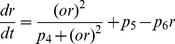
(1)

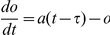
Based on the principles of mass action kinetics, these equations describe the production and degradation of the hormones ACTH (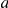) and CORT (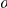), as well as glucocorticoid receptor (GR) density (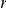) in the pituitary. The system is characterised by feed-forward and feedback connections: CRH acts on pituitary corticotroph cells to release ready-available ACTH, which in turn stimulates the synthesis and secretion of CORT from cells of the adrenal cortex. However, unlike ACTH which is pre-synthesised and stored within the pituitary, synthesis of CORT can only begin in the presence of ACTH. This results in a delayed response of the adrenal gland to ACTH. In addition to these feed-forward processes, the model also takes into account negative feedback by CORT (mediated by GR) at the level of the pituitary [14], [15]. Full details of the derivation of this model can be found in [7] and its accompanying supplementary material.

In deriving this model, we have made a number of assumptions. First, we assume that glucocorticoid feedback at the hypothalamus is not an important factor in regulating basal activity of the axis. Although there is good evidence suggesting that levels of corticosterone associated with the stress response can have rapid effects on hypothalamic activity [16], there is little evidence for rapid inhibition of hypothalamic activity by concentrations of glucocorticoids found in the normal basal state. Indeed, most of the current evidence supports the notion that the predominant site of action of glucocorticoids on basal HPA activity occurs at the level of the pituitary [17]–[19]. In light of this, we have assumed that basal activity of the axis is predominantly regulated by glucocorticoid negative feedback at the level of the anterior pituitary, and we therefore treat hypothalamic drive on the pituitary as a parameter (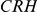) rather than a variable of the system. Further, we do not attempt to distinguish between a positive hypothalamic gain induced by CRH or AVP and without loss of generality lump these together into this single parameter 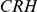. In addition to the CRH drive, there are six other parameters that determine the dynamical behaviour of the model. The parameters 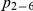 represent dimensionless forms of rate constants of the system, and the dimensionless parameter 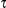 represents a discrete delay, which accounts for the delayed response of the adrenal gland to ACTH. Values of model parameters were chosen following the analysis in [7], such that in the unperturbed state there was an approximately hourly oscillation in ACTH and CORT. Specifically, 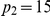, 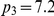, 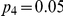, 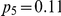, 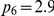, and 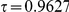 which corresponds to a delay of approximately 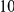 min in dimensional units (which is consistent with experimentally observed oscillations in ACTH and CORT). The key components of this model and the simplifying assumptions are illustrated in Figure 2.

To simulate the model computationally, we used a fourth-order Adams-Bashforth multistep integrator with a discretisation of 200 points for the delay period. Simulations were initially run for a sufficient length of time to allow for the decay of any transient behaviour. The time difference between the last two peaks was then used to calculate the endogenous period 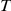 of the system. From this point on, simulations were resumed such that the dimensionless time 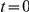 corresponded to the maximal value of an ACTH pulse.

### Modelling a stress input

**Figure 3:**
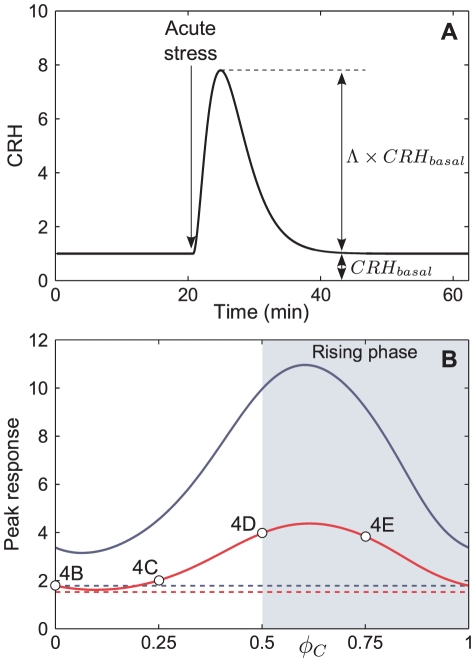
Timing of CRH-impulse determines magnitude of CORT response. (A) Profile of CRH-impulse where the amplitude 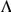 is scaled by the basal level of CRH. (B) Amplitude response curves of ACTH (solid blue) and CORT (solid red) computed for 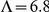 with varying phase 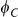 of the CRH impulse (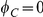 corresponds to the peak of the CORT pulse). As a reference, the maximum levels of basal oscillations in ACTH (dashed blue) and CORT (dashed red) are also plotted. The shaded region indicates values of 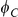 that correspond to the rising phase of the CORT oscillation. Markers on the CORT amplitude response curve correspond to the time histories plotted in Figure 4B–E.

To consider the effect of an acute stress, we perturbed the basal level of CRH with an impulse of the form 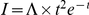, where the amplitude 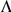 of the impulse is given as a multiple of the basal level of CRH (Figure 3A). This mathematical form was specifically chosen to capture known biological alterations in CRH following an acute stress; namely the sharp increase in CRH immediately following the onset of an acute stress, followed by a slower decay back to basal levels, due to the transient nature of the stress. Impulses in CRH were applied following a peak in CORT with relative phase 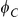, scaled by the endogenous period 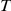. For a range of discrete values of 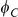 two quantities were computed: the amplitude response and the phase shift of subsequent peaks in CORT relative to the endogenous case. Computing these quantities across one period of the endogenous oscillation enabled us to calculate amplitude response information and also the phase response curve (PRC). The PRC is a natural tool to quantify how perturbations affect the dynamics of an oscillator and has been widely applied in the study of biological rhythms over a range of time-frames; from circadian cycles [20], [21], to more rapid neural oscillations [22]. It is a method for investigating the transient change in oscillation period resulting from a perturbation to the oscillator, and can be visualized by plotting the *normalized* phase 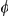 (or here, 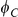) of the oscillator, against the resulting change in phase 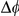.

## Results

### Mechanistic explanation of relationship between timing of stress and magnitude of CORT response

In the late 1990s, investigations into the effect of acute stress on the amplitude of the CORT response were performed using three strains of rat: female Sprague-Dawley (SD) [10], female Lewis [11], and male Piebald-Viral-Glaxo (PVG) with induced arthritis [12]. A consistent finding in all three studies was that the response to a stress applied during the *rising phase* of an endogenous CORT pulse was enhanced, relative to the response to a stress applied during the *falling phase*. Due to the nature of these studies, the timing of the stress could only be determined retrospectively upon comparison with the measured CORT levels, and further could only be determined to the nearest 10 min (the sampling interval).

We first used the data from these experimental studies to calibrate the model in terms of the amplitude of the CORT response to stress. For each experimental study, we computed the ratio between the maximum of the mean CORT response to a stress applied during the rising phase, and the maximum of the mean CORT response to a stress applied during the falling phase. The resulting ratios for the three studies were 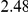 (SD), 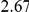 (Lewis), and 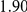 (PVG), which were averaged to give a final ratio of 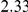. The amplitude 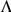 of the CRH impulse applied in the model was then tuned to match the ratio observed experimentally. Figure 3B shows the amplitude response curves of ACTH and CORT, where values of 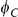 corresponding to the rising phase are indicated by the shaded region. As a reference, the maximum of the basal oscillations for ACTH and CORT are also shown as dashed lines. Taking the mean CORT response during the rising phase as the area between the amplitude response curve (solid red) and the endogenous maximum line (dashed red), and similarly for the falling phase, we found that a value of 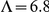 gave the correct ratio of 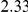.

**Figure 4:**
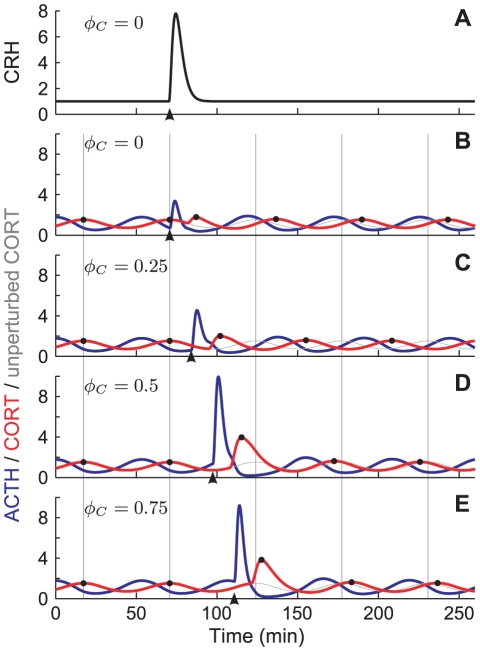
Computational illustrations of timing relationship between a CRH impulse and the magnitude of the CORT response. (A) CRH impulse corresponding to 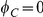 for 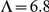. (B–E) Time histories showing of levels of ACTH (blue) and CORT (red) for fixed 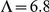 and values of 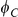 as indicated in the panels. Vertical arrow in each panel indicates the timing of the applied CRH impulse. Levels of CORT in the absence of an impulse are shown in grey, with expected peaks indicated by vertical lines. The induced phase shift is the time-separation between expected peaks (vertical lines) in the unperturbed case and the actual peak in CORT (black points) for the perturbed case.

To determine more explicitly the effect of the timing of the stress on the magnitude of the hormonal response, we computed individual ACTH and CORT time histories for a CRH stress impulse (with 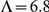) applied at four different values of the phase 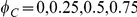 (Figure 4). Black arrows indicate the precise timing of the CRH impulse (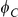), and peaks in CORT are indicated by black points. The grey curve shows a time history of CORT for the endogenous (unperturbed) case, with endogenous CORT peaks marked by vertical lines.

In each case, the CRH impulse is rapidly followed by an ACTH response, which is in turn followed by a response in CORT (Figure 4B–E). In line with experimental observations [23], the ACTH response consists of a more rapid activation and degradation phase in camparison to the CORT response. The most notable difference between the four cases is that the magnitude of the ACTH and CORT responses depends critically on the value of 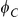
–that is, on the “timing” of the stress–which is also consistent with experimental findings [10]–[12]. The ACTH and CORT responses to a stress applied at phase 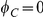 are barely noticeable relative to the peak levels of the endogenous oscillation (Figure 3B and Figure 4B). However, when a stress impulse coincides with the rising phase (e.g., 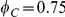) of the CORT oscillation (Figure 3B and Figure 4E), the amplitude of the response is considerably larger, both relative to a stress applied during the falling phase, and to the peak levels of the endogenous oscillation. From a dynamical systems perspective, when the stress perturbation coincides with the rising phase, the stress is acting in the same direction of motion as the endogenous oscillation, resulting in a pituitary-adrenal response that is significantly larger than the maximum of the basal oscillation. However, when levels are decreasing, the stress perturbation acts against the direction of motion of the endogenous oscillation. Thus, even a large amplitude impulse applied during the falling phase may only result in a limited response from the system.

### Stress shifts the ultradian rhythm in a phase-dependent manner

A further prediction from the model is that the stress impulses can induce phase shifts in the ultradian rhythm (see simulations in Figure 4B–E). This can be seen by comparing the timing of the last peak in CORT in the perturbed case (red) with the nearest peak in the unperturbed case (vertical lines). For example, for 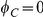, the phase is delayed and the peak in CORT comes after the unperturbed peak (the stress impulse moves *against* the direction of motion). Whereas for 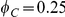 and 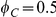, the phase is advanced and the peak in CORT is brought forward in time (the stress impulse moves *in agreement with* the direction of motion). At 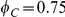 there is almost no change in phase. Thus, depending on the timimg of the stress (i.e., 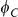), the phase of the ultradian rhythm can either be advanced or delayed.

**Figure 5:**
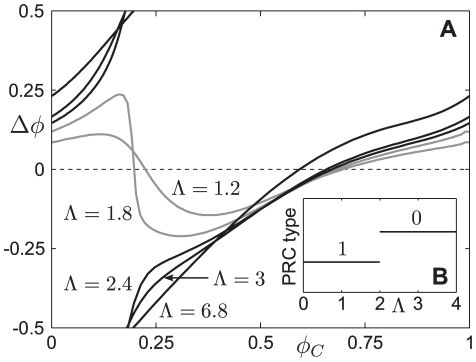
Parameter dependent profiles of phase response curves. (A) Phase response curves (PRCs) for different values of the stress impulse amplitude 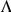 as indicated. For 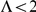 the model exhibits Type 1 phase-resetting (grey curves) with a sharp but continuous change in phase near 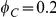. For 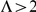 the model exhibits Type 0 phase-resetting (black curves) with a discontinuous change in phase near 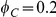. (B) Type of PRC curve plotted against 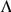.

If we define the phase shift 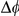 as the difference between the phase in CORT of the perturbed and unperturbed solutions, where a positive value of 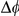 represents a phase delay, and a negative value a phase advance, then we can plot the relationship between 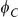 and 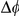 (Figure 5A). We can then further investigate how this phase response curve (PRC) for the system depends on the magnitude of the acute stress, defined by 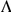. We computed 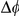 for 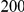 discrete values of 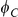 in the interval 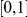 and for five different magnitudes of stressor (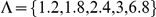) (Figure 5A; compare with the time histories in Figure 4B–E, which were computed with 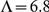 and phase values 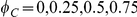).

For 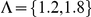 (grey curves) the phase response curves are smooth and pass through 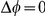 at 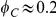. For 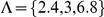 (black curves) the phase response curve has an apparent discontinuity where it passes through 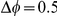 which coincides with 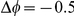 due to the periodic nature of 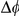. There is, in fact, a phase slip at 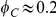 for these cases. The continuous PRCs that pass through 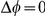 are classically known as Type 1, and the PRCs with a phase slip are known as Type 0 [20]. The inset panel Figure 5B shows that this qualitative change in the type of PRC curve occurs in our system for 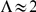. Note that in performing this analysis, we have assumed that the theory concerning perturbations to limit cycle oscillations, as applicable for systems without delays, is also applicable for our model that includes a single discrete delay term.

### Confirmation of predicted Type 0 phase-resetting mechanism from *in vivo* stress-response data

To explore the model prediction of a phase-resetting mechanism, we approximated the period of the endogenous CORT oscillations and determined the phase at which the 10 min stress was applied. In order to achieve this, we could only include a subset of all experimental data which satisfied the following conditions:

1. At least two clear pulses after application of the noise stress.
2. At least one clear pulse prior to application of the noise stress.

**Figure 6:**
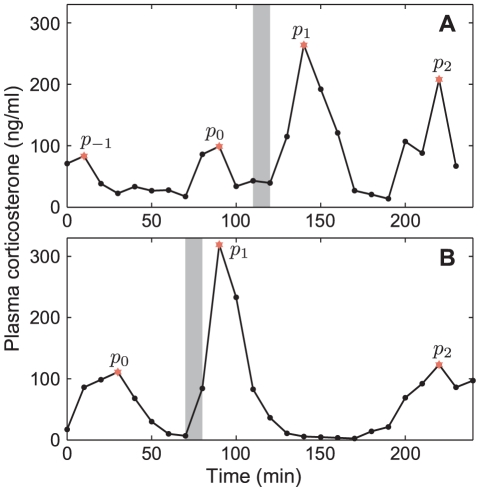
Determining phase information from experimental stress-response data. (A–B) Illustration of how peaks are selected in order to compute the phase information from experimental stress-response data. The time histories show levels of CORT sampled at 10 min intervals in exemplar female Sprague-Dawley (A) and female Lewis (B) rats. Shaded region indicates the period of the applied noise stress. Selected peaks (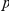) are marked red.

These conditions resulted in suitable data from 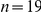 animals across the three studies [10]–[12]. Figure 6 presents two exemplars of the data we used. Specifically, the first condition enabled us to *approximate* the period 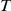 of the endogenous cycle as being the time interval between 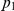 and 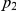. Whilst there is some variability in this frequency from pulse to pulse, typically this is of the order 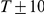 min. The second condition enabled us to approximate the relative position (the phase) on the endogenous period 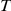 at which the noise stress was applied. For example in Figure 6A, 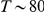 min and the 10 min stress (shaded region) is applied approximately 20 min after 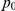. This corresponds to a relative phase 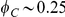. Finally, we determined the magnitude of the phase shift by considering the time interval between 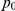 and 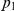 relative to the endogenous period 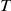.

**Figure 7:**
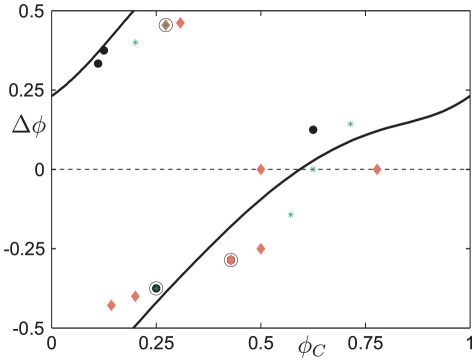
Comparison of theoretical PRC with experimental data confirms a Type 0 phase resetting mechanism. The Type 0 phase response curve for 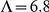 as computed with the model (black curve). The experimental data, plotted at discrete points, is shown for eight female Sprague-Dawley rats (red diamonds), five female Lewis rats (black dots), and six male PVG rats (green stars). Points where two samples take the same value are circled.

We computed the phase resetting information for all the individual time histories (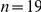) in the experimental data set (Figure 7). Where the data points from more than one time history coincide, the point is circled in black. The PRC computed from the mathematical model with the previously determined stress impulse amplitude (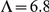) is also shown. Interestingly, the data appears consistent with a Type 0 PRC with a phase slip close to 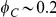, representing the “transition point” between an apparent phase advance and phase delay of the endogenous oscillation.

We then tested whether this close agreement between the experimental data and the theoretical PRC could have occurred by chance. To consider this, we calculated the goodness of fit between the theoretical PRC and the values extracted from the experimental study, using least squares to estimate the Euclidian distance between the theoretical curve and experimental data. Employing bootstrap statistics resulted in 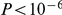 when comparing the fit of the experimental data with fits obtained by choosing 19 randomly selected phase shifts with equivalent phase positions to those of the experimental data.

## Discussion

Basal activity of ultradian glucocorticoid secretion depends on many factors including genetic and epigenetic status as well as the physiological state of the animal [4]. A further very important modulator of rhythmic glucocorticoid secretion is the response to a stressor. Since dynamic changes in glucocorticoid levels in tissue parallel those in plasma [24], and pulsatile activation of GR has recently been shown to be crucial for normal gene transcription [2], [3], any disruptions to the glucocorticoid rhythm will also be sensed at target sites and will have significant repercussions at the level of receptor signalling. It is therefore very important to understand the dynamic interaction between stress-induced hypothalamic activity and the endogenous glucocorticoid rhythm.

Recent theoretical modelling of this system suggests that the ultradian glucocorticoid rhythm is not solely controlled by pulsatile hypothalamic activity, but is primarily generated by a dynamic systems-level sub-hypothalamic oscillator involving interactions between the the anterior pituitary and adrenal cortex [7]. Based upon this hypothesis, we used the theory of phase response curves to explain the effects of timing on the magnitude of the CORT response to stress as had been consistently observed experimentally. Whilst phase response curves have been used to study the effects of perturbations on low frequency oscillatory activity (e.g., circadian rhythms [20]) or very high frequency activity (e.g., neural firing [22]), their use to characterize oscillating systems at ultradian frequencies is less common. However, neuroendocrine systems typically encode information in this intermediate frequency regime and PRCs provide a valuable, natural tool with which to study the effects of exogenous perturbations to these hormone systems that are endogenously rhythmic.

In addition to explaining earlier observations that the magnitude of the stress response depends on the timing of the stress, our modelling work further predicted that an external stress can act as a resetting mechanism to the phase of the endogenous ultradian rhythm. Using the experimentally estimated value of the amplitude of the external stress within our model, we observed a Type 0 phase response curve which accurately predicted the type of response observed across the three experimental studies. It is natural to ask what, if any, significance we should ascribe to a phase response curve of the type we have validated from the experimental observations?

**Figure 8:**
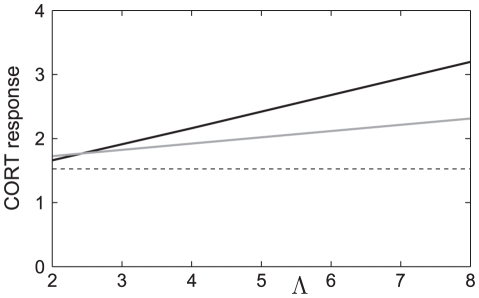
Comparing the CORT response to an acute stress in the oscillatory and non-oscillatory regimes. Basal CRH is set such that basal CORT in the non-oscillatory case matches the maximum level of CORT for the oscillatory case (dashed black). In the non-oscillatory case the response to a stress is independent of the timing of the stress (grey), whilst for the oscillatory case we present the averaged response to an incoming stress applied at every point over a period of oscillation (solid black). 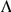 represents the magnitude of the stress. For small stressors, the response in both cases is comparable, whilst for larger stressors the response in the oscillatory case is significantly greater. For comparison, the amplitude of the acute noise stress was estimated to be 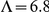, for which case we see a much greater response within the oscillatory regime.

We hypothesise that this endogenous oscillatory activity has evolved for two main reasons. Firstly, the level of CRH drive required to generate a steady-state in CORT at a level equivalent to the peak of a pulse is nearly four times greater than the level required for generating the pulsatile pattern (i.e., 93 as opposed to 25). Secondly, the ability to differentially respond to perturbations is much greater when in the oscillatory regime, compared to the system in equilibrium. To illustrate this, we considered the amplitude response in CORT resulting from a stress perturbation when the system was either in the steady-state or oscillatory regime. In the oscillatory case, we averaged the amplitude response across all values of 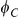. We then considered these values for a range of stress amplitudes 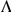 (Figure 8). We found that for the value of 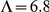 estimated from the data, the average response is approximately 50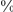 larger than the response when the system is in equilibrium. Moreover, as the value of 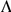 decreases (corresponding to smaller stress inputs to the system), the average value of the response drops towards the maximum value of the basal oscillation in CORT (dashed line). This provides a mechanism through which the system can effectively filter out low-amplitude stochastic perturbations from the internal and external environment, but remain markedly responsive to more significant perturbations (i.e., stressors).

In conclusion, our findings provide further evidence to support the hypothesis of a systems-level sub-hypothalamic oscillator that is responsible for the generation of ultradian glucocorticoid pulsatility. They further suggest that this rhythmicity of the pituitary-adrenal network governs hormonal *responsiveness* to stress, and the coupling between this and stress-induced hypothalamic inputs is what determines the hormonal *stress response*. Intriguingly, whilst chronic stress results in long-term changes in dynamic rhythmicity (as witnessed from the loss of a circadian rhythm in PVG rats with chronic inflammatory stress; see Figure 1C), the response to acute stress is consistent in both the PVG model and the wild-type animals. This suggests the existence of two distinct mechanisms whereby chronic stress regulates hormonal responsiveness, whilst acute stressors regulate the hormonal response.
